# Research studies on the bioactive compound profile of non-timber products from the Colombian Orinoquia region

**DOI:** 10.3389/fnut.2026.1971878

**Published:** 2026-10-21

**Authors:** J. Mauricio Peñarrieta, Daniela Miranda-Flores, Erika Leonor Zambrano-Moreno, Jhon Edinson Nieto-Calvache, Leonardo A. Alonso-Gómez

**Affiliations:** 1 Instituto de Investigaciones Químicas IIQ, Universidad Mayor de San Andrés UMSA, La Paz, Bolivia; 2 Grupo de Investigación “Ciencia, Tecnología e Innovación Agroindustrial (CITIA)”, Universidad de los Llanos, Villavicencio, Colombia

**Keywords:** antioxidant capacity, *Caryodendron orinocense*, Colombia, Copaifera spp., *Mauritia flexuosa*, Orinoquia

## Abstract

**Introduction:**

The Colombian Orinoquia region is home to a wide variety of non-timber forest products (NTFPs) with the potential to stimulate bioeconomic development. This study evaluated the bioactive compound profile and viscoamylographic properties of three plant materials: cacay (Caryodendron orinocense), copaiba (Copaifera spp.), and moriche (*Mauritia flexuosa*).

**Methods:**

Different plant matrices (flours, leaves, and oils/oleoresins) were evaluated. Total phenols, flavonoids, and antioxidant capacity (ABTS and FRAP) were quantified, pasting behavior was analyzed, and principal component analysis (PCA) was performed.

**Results:**

Cacay flour showed moderate gelatinization (peak viscosity: 98 BU; setback: 116 BU), suggesting use as a stabilizer in food systems. Moriche mesocarp (OMF) and endocarp flours (WEF) exhibited negligible gelling capacity but high antioxidant activity (ABTS: 17–19 µmol TE/g; FRAP: 18–20 µmol TE/g), despite lower phenolic content (12–15 µmol GAE/g). Cacay leaves had the highest phenolic content (28.9 µmol GAE/g) and antioxidant capacity (ABTS: 14.8 µmol TE/g; FRAP: 16.1 µmol TE/g). Copaiba oleoresin exhibited greater phenolic content (3.11 µmol GAE/L) and antioxidant capacity than cacay oil. Principal component analysis revealed an inverse relationship between technological functionality and antioxidant composition.

**Discussion:**

These findings underscore the potential of cacay as a functional flour and moriche as a source of functional and bioactive compounds, providing a scientific foundation for their valorization in the food, cosmetic, and pharmaceutical industries.

## 1 Introduction

Globally, the transition toward a bioeconomy is driving the replacement of fossil-based resources with renewable biomass as a strategic response to the climate crisis and the demand for sustainable production models. In this context, tropical regions harbor vast bioeconomic potential in their non-timber forest products (NTFPs), which serve as a key alternative for agro-industrial development and ecosystem conservation (1, 2). In Colombia, the Orinoquia region stands out as a reservoir of plant megadiversity with immense potential that remains largely unexplored scientifically (3). Although NTFP use in this region has historically relied on ancestral knowledge, agro-industrial valorization enables their transformation into functional ingredients and high-value-added processed matrices. Previous studies have begun to reveal the phytochemical potential of wild and cultivated species in the region. In the case of cacay (*Caryodendron orinocense* H. Karst), literature reports that its oil is rich in linoleic acid and tocopherols, while extraction by-products, such as residual cake flour, retain significant concentrations of phenolic compounds and high-value proteins (4, 5). Regarding copaiba (Copaifera spp.), several studies across the Amazon basin have characterized its oleoresin as containing sesquiterpenes and diterpenic acids with recognized anti-inflammatory, antimicrobial, and antioxidant activities (6, 7). Likewise, in moriche, also known as *“palma real”* in Bolivia (*Mauritia flexuosa*), the fruit mesocarp has been documented as an exceptional source of carotenoids (β-carotene), vitamin C, and polyphenols that can neutralize free radicals and protect against oxidative stress (8). However, most studies reported in the literature have focused on ecotypes from the Brazilian or Peruvian Amazon. Consequently, information on the composition and bioactivity of NTFPs specifically from the Colombian Orinoquia is scarce, where distinct soil and agroclimatic conditions can significantly alter plant metabolomic profiles. Furthermore, knowledge gaps persist regarding the comprehensive characterization of underutilized fractions, such as cacay press cake flour or differentiated mesocarp and endocarp flours from moriche, and their viscoamylographic behavior, a critical techno-functional parameter for evaluating their performance in food and industrial formulations (9, 10). Therefore, this study aimed to evaluate the bioactive compound profile (total phenols, flavonoids, and antioxidant capacity via ABTS and FRAP) and the viscoamylographic properties of matrices derived from NTFPs of the Colombian Orinoquia: cacay (oil and cake flour), copaiba (oleoresin), and moriche (mesocarp and endocarp flours), establishing a scientific foundation for their technical and industrial valorization in the agro-food and cosmetic sectors.

## 2 Methodology

### 2.1 Sample collection

This study examined dried cacay leaves (CL), cake meal (CF) from cacay oil production, and cacay oil (CO) from *Tacay Natural Oils*. Copaiba Oleoresin (COR) was obtained from the company *VitaCopaiba*. All samples were collected from the Orinoquia region of Colombia. The CLs were collected from trees located at the Universidad de los Llanos; they were selected, washed, disinfected, and dried at 50 °C in a Memmert UF110 forced-convection oven. (Memmert GmbH + Co. KG, Schwabach, Germany) until the moisture content stabilized. The CLs were then ground and stored under vacuum until use (11).

From the moriche, an orange-colored mesocarp and a white, parchment-like endocarp were obtained. These samples were obtained manually, allowing the pulp to be separated from the inner portion of the fruit. First, the whole ripe fruits were washed and sanitized to remove surface impurities. They were then blanched at 80 ± 5 °C to facilitate exocarp removal; once removed, the fleshy orange mesocarp (the edible pulp) was obtained. Finally, the white, parchment-like endocarp surrounding the seed was exposed. This portion was isolated manually by separating the inner layer from the pit. The two resulting samples, one obtained from the orange-colored mesocarp (OMF) and the other from the white-colored endocarp (WEF), were washed, drained, and dried in a Memmert UF110 forced-convection oven (Memmert GmbH + Co. KG, Schwabach, Germany) at 40 °C until a constant moisture content is reached, for subsequent grinding and storage at room temperature (12).

### 2.2 Viscoamylographic analysis

The rheological behavior of flours in the presence of water and under a controlled thermal cycle was evaluated using viscoamylography with a Brabender-type viscosity analyzer. The preparation of an aqueous suspension of each sample was conducted in accordance with a standardized concentration, as outlined below: For instance, 5 g of flour made from the white endocarp of the moriche palm, 10 g of flour made from the orange mesocarp of the moriche palm, and 15 g of Cacay flour were meticulously measured and diluted in a 100-mL distilled water solution. The mixture was then placed in the instrument’s vial. The analysis followed a thermal program that included: (1) Initial heating started at 30 °C and increased to 85–95 °C at a constant rate. (2) Holding at the maximum temperature for a specified time. (3) Controlled cooling to 50 °C, followed by a final stabilization period.

Throughout the cycle, the equipment continuously recorded viscosity in Brabender units (BU), allowing identification of the main viscoamylographic parameters: onset of gelatinization, peak viscosity, breakdown, final viscosity, and setback. These parameters provided information on the thermal stability of the flours, their shear resistance, and their tendency to retrograde during cooling. The measurements were taken using a Brabender Micro Visco Amillograph (Essen, Germany).

### 2.3 Measurement of total antioxidant capacity

Before analysis, 100 mg of sample was weighed. The mixtures were sonicated at 0 °C for 15 min. They were then centrifuged at 12000 rpm for 2 min to decant the supernatant, which was stored at 4 °C. Extraction for total antioxidants was performed in triplicate to obtain representative data (13, 14).

For lipid-rich matrices (cacay oil, CO, and copaiba oleoresin, COR), samples were first subjected to phase extraction to ensure solubilization in aqueous assay conditions. Specifically, 1.0 g of each lipid sample was dissolved in 10 mL of ethanol (95%) and vortexed for 2 min. The ethanolic extract was subsequently diluted with distilled water (1:1, v/v) to yield a miscible solution suitable for spectrophotometric determinations. This preparation ensured that phenolic compounds and other polar constituents in the lipid fraction reacted with Folin–Ciocalteu, Total Flavonoids determination reagents, ABTS, and FRAP reagents. The density was determined following ISO 6883:2017 to obtain results on a dry-weight basis for better data comparison.

Total antioxidant capacity was measured by the ABTS [2,2′-azino-bis(3-ethylbenzothiazoline-6-sulphonic acid)] method and a modified FRAP method (Ferric reducing antioxidant power). Both are spectrophotometric methods, and the absorbance readings were performed using an Ultrospec 3,000 (Pharmacia Biotech, Uppsala, Sweden) at 25 °C. Trolox (6-hydroxy-2,5,7,8-tetramethylchroman-2-carboxylic acid) was used as the standard compound. A stock solution of Trolox (5 mmol/L) in ethanol was stored at −20 °C.

The colorless ABTS (7 mmol/L) was oxidized to a green ABTS^•^ radical cation by the addition of potassium persulfate (2.42 mmoL/L) and kept for 12–16 h at room temperature in the dark. The reagent was stable for 2–3 days when stored in the dark. On the day of analysis, dilute the ABTS• + solution with ethanol to an absorbance of 0.70 (±0.02) at 734 nm. After adding 1.0 mL of ABTS• + solution to 100 μL of the sample, the mixture was stirred for 30 s, and the absorbance at 734 nm at 25 °C was recorded for 6 min. The decrease in absorbance upon addition of the sample was compared with that of a standard curve prepared with Trolox (20–200 μmoL/L) (15, 16).

In the FRAP method, electron-donating substances reduce the yellow Fe^3+^-TPTZ complex to the blue Fe^2+^-TPTZ complex under acidic conditions. Any electron-donating substance with a half-reaction redox potential lower than that of Fe3+/Fe2 + TPTZ will drive the formation of the blue complex forward. The FRAP reagent was a mixture of 0.1 mol/L sodium acetate buffer (pH 3.6), 10 mmol/L TPTZ, and 20 mmol/L ferric chloride (10:1:1, v/v/v). Add 900 μL of reagent, 90 μL of water, and 30 μL of sample. Start absorbance readings immediately after adding the sample and record at 593 nm for 10 min. The blank consisted of 120 μL of water and 900 μL of reagent. The final absorbance of each sample was compared with that of a standard curve made using Trolox (100–1,000 μmoL/L). The data were expressed as μmol Trolox equivalents per gram of dry matter (14, 17).

### 2.4 Measurement of total phenolic compounds

The total phenolic compounds (TPC) were determined using the Folin–Ciocalteu reagent, which oxidizes phenols to phenolates at alkaline pH in a saturated sodium carbonate solution, yielding a blue molybdenum-tungsten complex. The Folin–Ciocalteu reagent (diluted 10-fold; 2.5 mL), 2 mL of saturated sodium carbonate (75 g/L), and 50 μL of sample (diluted 10-fold) were mixed, homogenized for 10 s, and heated for 30 min at 45 °C. The absorbance at 760 nm was read after cooling to room temperature. The absorbance of each sample was compared with that obtained from the standard curve made from gallic acid (235–1,176 μmoL/L). The data were expressed as μmol gallic acid equivalents per gram of dry matter (15, 18–20).

### 2.5 Measurement of total flavonoids

The total flavonoid content was determined according to Jia et al. (21). The sample was mixed with a reagent containing aluminum chloride and sodium nitrite, forming a pink flavonoid-aluminum complex in an alkaline medium. A solution corresponding to 30 μL of sodium nitrite (10%), 60 μL of aluminum chloride hexahydrate (20%), 200 μL of NaOH (1 M), and 400 μL of water was added to 100 μL of each sample. Absorbance readings at 510 nm were collected 5 min after adding the sample and recorded every 20 s for 1 min. A reagent blank containing water instead of the sample was used. The final absorbance of each sample was compared with a standard curve made from catechin (69–689 μmoL/L). The data were expressed as μmol catechin equivalents per gram of dry matter (15, 20).

### 2.6 Statistical analysis

The results are expressed as the mean of three independent replicates ± the standard deviation. Data processing and statistical analysis were performed in RStudio (version 2026.04.526).

## 3 Results

### 3.1 Viscoamylography of flours

Table 1 compares the viscoamylographic parameters of wheat flour (reference standard): CF, OMF, and WEF. The wheat values correspond to ranges reported in the technical literature, while the others were obtained experimentally. The table allows for an evaluation of the functionality of these ingredients as gelling agents or thickeners.

**Table 1:** Pasting profiles of the cacay-pressed cake and moriche fractions.

Sample	Peak viscosity (BU)	Final viscosity (BU)	Pasting temperature (°C)	Setback viscosity (BU)
Wheat flour (ref.)	400–600	500–700	60–65	100–200
CF	98	184	~82.3	116
OMF	4	0	~33.2	0
WEF	0	1	~30.4	0

CF, Cacay Cake Flour; OMF, flour made from the orange mesocarp of the moriche palm; WEF, flour made from the white endocarp of the moriche palm.

CF exhibits clear gelation, with viscosity increasing as it cools. The high setback (increase in viscosity upon cooling) suggests good retrogradation capacity, which is desirable in structured products, such as gels or baked goods, that need to retain their shape. However, the maximum viscosity (98 BU) is moderate to low compared to raw quinoa (1,197 BU) or the oca flour (577 BU) (22). Therefore, its thickening capacity is limited but stable. This performance profile suggests potential applications in products with a soft texture, gels, and baked goods.

OMF exhibits minimal viscoamylographic activity: it does not develop viscosity upon heating or cooling. Gelatinization occurs at a very low temperature, but the starch does not form a gel or any structure. This profile suggests that it either contains no starch or that the starch has been degraded, and it is not useful as a thickening agent or gelling agent. Therefore, it cannot be used as a stabilizer or thickener in food products. WEF behaves similarly to OMF; it does not develop significant viscosity. It does not exhibit any relevant functional activity as a starch. Therefore, its use as a thickener or gelling agent is not feasible without prior modification.

This behavior is consistent with previous reports which indicate that starches subjected to structural degradation or unconventional drying methods often fail to develop viscosity or gelation capacity. Liu et al. (23) described non-crystalline starches with low processing viscosity, while Jiménez-Guzmán et al. (22) and Köksel et al. (24) reported that freeze-drying or lyophilization can disrupt molecular order, thereby reducing retrogradation and pasting properties. Similarly, Meadows (25) observed that degraded starch fractions in rice flour can eliminate RVA peaks. The findings support the conclusion that moriche fractions, in their native state, lack functional starch properties and require modification to be used as thickeners or stabilizers.

CF has a moderate functional profile, suitable for applications that require a smooth texture and stability during cooling. OMF and WEF, in their current form, do not function as gelling starches; therefore, consider enzymatic modification or use as a nutritional or flavoring ingredient (rather than a functional ingredient). In comparison, oca and quinoa exhibit viscoamylographic profiles that are much more useful for formulating functional or food products that require viscosity, thickness, or structure (22).

When benchmarked against wheat flour, a well-established theoretical model with high peak and final viscosities, a moderate pasting temperature, and pronounced setback suitable for baking systems, the experimental samples exhibited distinct functional behaviors. Cacay starch showed an intermediate pasting profile, suggesting potential use in systems requiring gentle gelation or stability upon cooling. In contrast, neither moriche sample displayed useful viscoamylographic properties in its native state (22).

### 3.2 Phenolic compounds, flavonoids, and the antioxidant capacity of cacay leaves, their press cake, and moriche fractions

In terms of magnitude, the values obtained are consistent with recent literature: the phenolic content of OMF (14.69 ± 0.72 μmol GAE/g) and WEF (12.25 ± 0.41 μmol GAE/g) is in the same order of magnitude as that reported for moriche seeds from the Amazon (15.87 μmol GAE/g) (26, 27). The phenolic content of cacay flour (8.96 ± 1.46 μmol GAE/g) is nearly five times higher than that reported for oil (1.92 ± 0.04 μmol GAE/g) and cacay butter (1.77 ± 0.03 μmol GAE/g). This confirms that some phenolic compounds remain in the solid residue from pressing and do not migrate into the oil phase. However, its low antioxidant activity of 0.67 ± 0.10 μmol TE/g by ABTS and 2.85 ± 0.22 μmol TE/g by FRAP shows that a proportion of its phenolic compounds are present in bound forms with low reactivity, and that its antiradical activity is attributed to fat-soluble antioxidants, primarily tocopherols, that are concentrated in the oil fraction during pressing (5).

The CL had the highest phenolic content (28.87 ± 2.90 μmol GAE/g), about twice that of the moriche fractions (12.25 ± 0.41–14.69 ± 0.72 μmol GAE/g) and three times that of the flour in the CF (8.96 ± 1.46 μmol GAE/g). However, this order was not maintained when antioxidant capacity was assessed using the ABTS and FRAP methods. The moriche fractions, which had about half the phenolic content of the CF, significantly outperformed the CF in both ABTS (18.25 ± 1.71 vs. 0.67 ± 0.10 μmol ET/g P. S.) and FRAP (19.41 ± 2.09 vs. 2.85 ± 0.22 μmol ET/g P. S.). This behavior is consistent with Bensaada et al. (26): the ability of phenolic compounds to scavenge free radicals depends not on quantity, but on their chemical structure and on rearrangement or interconversion following extraction. This is also explained by the fact that the Folin–Ciocalteu method, when performed in an alkaline medium, quantifies phenolic compounds from cacay leaves that are possibly bound to cell wall polysaccharides, which contribute to the antioxidant response to a lesser extent than expected by the ABTS and FRAP methods without prior alkaline hydrolysis (28, 29). On the other hand, CF has a moderate phenolic content but almost no antioxidant activity, consistent with the low reactivity of its bound phenolics; in contrast, the moriche fractions, despite their lower phenolic content, exhibit high antioxidant activity, suggesting the contribution of other non-phenolic compounds, such as carotenoids and ascorbic acid, which are characteristic of this raw material (8, 30).

The flavonoid content was low for all samples (1.03 ± 0.17–4.37 ± 0.40 μmol EC/g P. S.) and much lower than the total phenolic compound content, indicating that the functional potential of the samples is dominated by phenolic acids rather than flavonoids. This finding is consistent with Rudke et al. (31), who identified protocatechuic acid as the major phenolic compound. OMF had the highest flavonoid content (4.37 ± 0.40 μmol GAE/g), which may be explained by the orange pigmentation of its flour. CF had the lowest values in the entire study for both phenolic compound content (8.96 ± 1.46 μmol GAE/g) and antioxidant capacity (0.67 ± 0.10 μmol ET/g in ABTS and 2.85 ± 0.22 μmol ET/g in FRAP). This result is consistent with previous findings that the antioxidant compounds in cacay are predominantly fat-soluble (tocopherols and tocotrienols) and migrate into the oil fraction during oil extraction. Furthermore, cacay cake meal stands out for its protein (34.8%), lipid (12.5%), and dietary fiber (20.1%) content, highlighting that its value stems more from its nutritional and techno-functional benefits than from its antioxidant activity (4).

### 3.3 Polyphenol and flavonoid content and the antioxidant capacity of copaiba oleoresin and cacay oil

Table 2 presents the TPH and antioxidant capacity of Copaiba oleoresin (COR) and Cacay oil (CO). The density was measured using the ISO 6883:2017 method. COR exhibited a TPH of 3.11 ± 0.15 μmol GAE/L, higher than that of the oil (1.47 ± 0.67 μmol GAE/L), as well as greater antioxidant capacity as assessed by the ABTS (0.76 ± 0.08 vs. 0.39 ± 0.02 μmol ET/L) and FRAP (6.45 ± 0.66 vs. 4.81 ± 0.09 μmol ET/L) assays. The TF content was slightly higher in the oleoresin (1.35 ± 0.86 μmol CE/L) than in the oil (1.09 ± 0.06 μmol CE/L). This behavior is consistent with the nature of both matrices. Cacay oil is rich in linoleic acid (85.6%) and contains a smaller non-saponifiable fraction (8.1%) (5, 32). Since oleoresin is a more concentrated extract, it retains a higher proportion of the polar and resinous fraction that carries phenolic compounds, which explains the observed enrichment. The direct relationship between phenolic content and antioxidant capacity is consistent with reports for this species and with the general framework of antioxidant capacity assays (33). The higher FRAP values compared to those of the ABTS assay are attributable to the different mechanisms and stoichiometries of the two assays: electron transfer in the FRAP assay vs. a mixed mechanism in the ABTS assay (34).

**Table 2:** Total phenolic and flavonoids of resin and oil.

Sample	TPH		TF		ABTS		FRAP	
	[μmol GAE/L]	[μmol GAE/g]	[μmol CE/L]	[μmol CE/g]	[μmol ET/L]	[μmol ET/g]	[μmol TE/L]	[μmol ET/g]
COR	3.11 ± 0.15^a^	2.94 ± 0.14^a^	1.35 ± 0.86 ^a^	1.27 ± 0.81^a^	0.76 ± 0.08 ^a^	0.71 ± 0.06^a^	6.45 ± 0.66 ^a^	6.08 ± 0.62 ^a^
CO	1.47 ± 0.67 ^b^	1.34 ± 0.61 ^b^	1.09 ± 0.06 ^a^	0.99 ± 0.05 ^a^	0.39 ± 0.02 ^b^	0.35 ± 0.02 ^b^	4.81 ± 0.09 ^b^	4.39 ± 0.08 ^b^

Different letters in the same column indicate significant differences (Tukey, *p* ≤ 0.05). In the second column, we measured density for COR and CO. The density for CO was 0.944 ± 0.002, and for COR it was 0.912 ± 0.001 at 20 °C, measured by the ISO 6883:2017 method.

### 3.4 Principal component analysis

To obtain a comprehensive overview of the antioxidant quality and techno-functional behavior of the flours (CF, OMF, and WEF), principal component analysis (PCA) was applied. This multivariate analysis tool is useful for characterizing botanical samples, as it correlates variables across principal components with minimal information loss. This facilitates identifying patterns of similarity and difference that are not always evident in traditional statistical analyses (35).

Figure 1 shows the resulting biplot, where the first dimension (Dim1) accounts for 97.1% of the total variance; thus, this component largely describes the data structure and allows us to identify the effects of the measured variables (35). It is important to acknowledge the limitations of this PCA, which was conducted on only three flour matrices (CF, OMF, WEF). Consequently, the interpretation applies only to these samples, limiting the generalizability of the statistical inferences.

**Figure 1:**
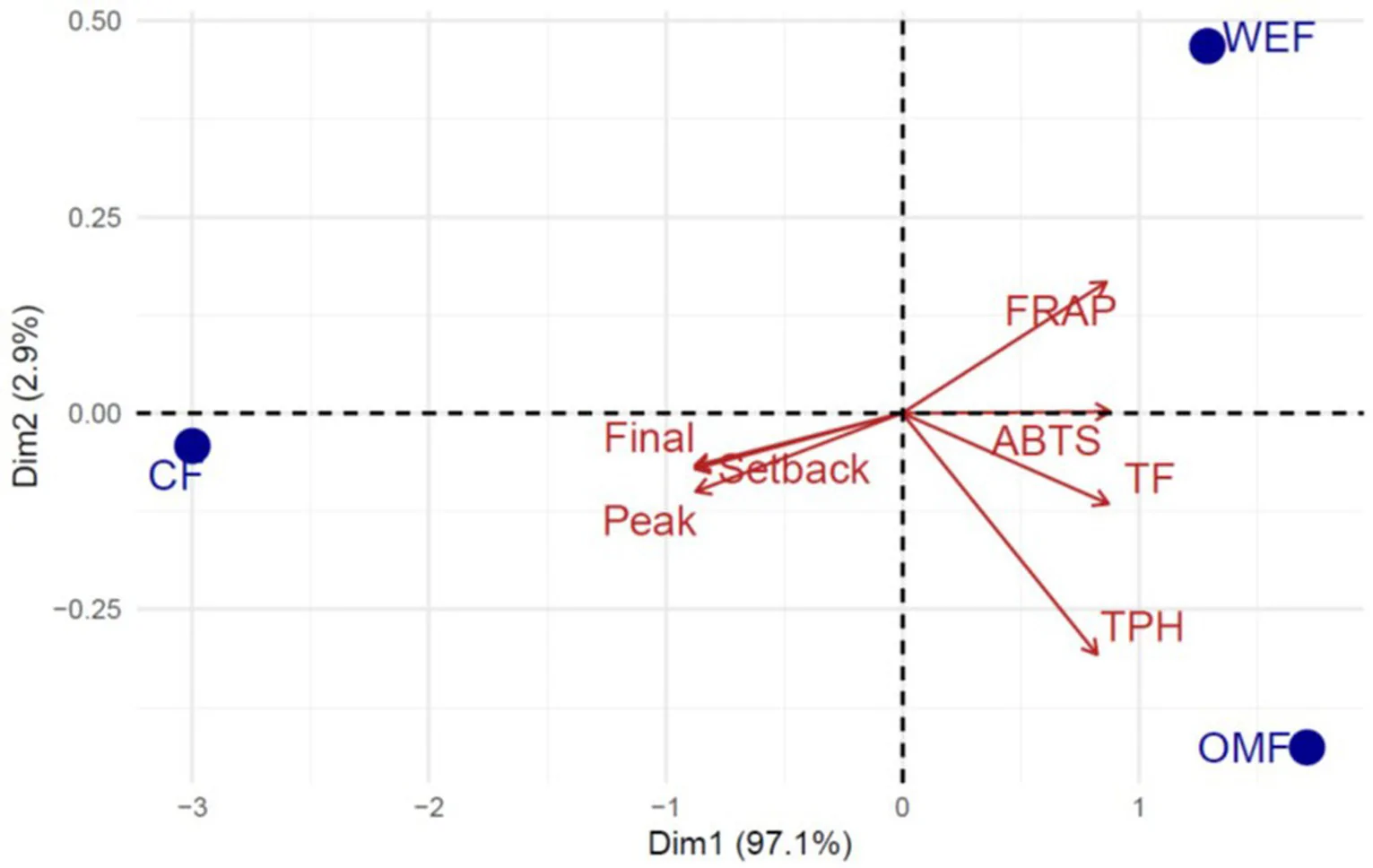
Principal component analysis (PCA) of the antioxidant capacity of bioactive compounds and the viscoamylographic profile in cacay and moriche flours.

Cacay cake flour (CF) was located in the negative quadrant of PC1, strongly associated with the viscoamylographic vectors (Peak, Final, and Setback viscosity), defining its profile as a thickening and structural agent, consistent with the parameters in Table 1. At the opposite end of the spectrum, moriche flours (OMF and WEF) clustered around the vectors for phenolic compounds (TPH), flavonoids (TF), and antioxidant capacity (ABTS and FRAP), reflecting their higher bioactive-compound content (Table 3). The marked collinearity among the TPH, TF, ABTS, and FRAP vectors reflects the well-known association between phenolic content and antioxidant response in plant matrices, mechanistically underpinned by the electron transfer common to these assays (26).

**Table 3:** Phenolic compounds, flavonoids, and antioxidant capacity of cacay leaves, their pressed cake, and moriche fractions.

Sample	TPH [μmol GAE/g]	TF [μmol CE/g]	ABTS [μmol TE/g]	FRAP [μmolTE/g]
CL	28.87 ± 2.90^a^	3.66 ± 0.76^b^	14.81 ± 1.99^b^	16.14 ± 1.93^b^
CF	8.96 ± 1.46^c^	1.03 ± 0.17^c^	0.67 ± 0.10^c^	2.85 ± 0.22^c^
OMF	14.69 ± 0.72^b^	4.37 ± 0.40^a^	19.05 ± 1.65^a^	18.35 ± 2.59^a^
WEF	12.25 ± 0.41^b^	3.63 ± 0.24^b^	17.45 ± 1.76^a^	20.47 ± 1.59^a^

CL, Cacay leaves; CF, Cacay flour; OMF, Flour made from the orange mesocarp of the moriche palm; WEF, White flour from the endocarp of the moriche palm. Different letters in the same column indicate significant differences (Tukey, *p* ≤ 0.05).

The opposing relationship between the viscoamylographic vectors and the antioxidant compounds and activity on Component 1 indicates an inverse relationship between technological functionality and antioxidant composition: samples with greater thickening power have the lowest phenolic content and antioxidant capacity (CF), whereas samples with higher phenolic content lack thickening power (WEF and OMF). This pattern confirms that botanical origin determines the behavior of the different samples and allows moriche to be classified as a source of bioactive compounds of interest to the cosmetic or pharmaceutical industry, and cacay as a sample of technological interest with potential applications in flour enrichment (5, 35).

## 4 Conclusion

This study comprehensively characterized the functional and bioactive properties of non-timber forest products from the Orinoquia region, specifically those derived from cacay and moriche, through viscoamylographic analysis and the determination of phenolic compounds, flavonoids, and total antioxidant capacity.

Rheologically, cacay starch showed a well-defined viscoamylographic profile, with moderate peak viscosity and a high setback value, indicating adequate retrogradation during cooling. This behavior suggests its potential for use in food products that require a smooth texture, thermal stability, and consistency after cooling, such as gels, baked goods, or semisolid formulations. In contrast, the moriche samples, both orange and white, showed little viscosity development or effective gelatinization, indicating limited functionality as a thickening or gelling starch in their native form.

A comparison with conventional flours, such as wheat flour, showed that cacay has intermediate functionality, lower than wheat but higher than moriche, which opens opportunities for its use as a complementary or alternative ingredient in gluten-free or functionally oriented formulations. Moriche, by contrast, should be prioritized for its nutritional and bioactive value rather than its rheological properties, or it should undergo technological modification to improve its functionality.

A limitation of this study is the lack of starch composition and structural analysis, which limits mechanistic interpretation of the negligible pasting viscosity observed in moriche flours. Future work should include starch quantification and microstructural characterization to better explain the viscoamylographic behavior.

Regarding the bioactive profile, the results showed that cacay leaves exhibited higher antioxidant capacity and higher levels of phenolic compounds and flavonoids than the seeds, highlighting the importance of utilizing traditionally underutilized plant byproducts. In moriche flours, phenolic compounds were more abundant than flavonoids, and antioxidant capacity varied by method (ABTS and FRAP), suggesting compounds with different antioxidant mechanisms of action.

Taken together, these results confirm the high potential of the NTFPs studied as sources of bioactive compounds and as raw materials for developing functional ingredients. From a bioeconomy and sustainability perspective, valorizing cacay and moriche supports the comprehensive use of tropical biodiversity and lays the scientific foundation for their application in the food, functional, and cosmetics industries.

## Data Availability

The raw data supporting the conclusions of this article will be made available by the authors, without undue reservation.
